# Supplementary material for: A Low to Medium-Shear Extruded Kibble with Greater Resistant Starch Increased Fecal Oligosaccharides, Butyric Acid, and Other Saccharolytic Fermentation By-Products in Dogs
Source: Microorganisms. 2021 Nov 4;9(11):2293. doi: 10.3390/microorganisms9112293 (PMC8621988; doi:10.3390/microorganisms9112293)
Supplement: Supplementary file 1 [file microorganisms-09-02293-s001.zip › microorganisms-1430430-supplementary.pdf]

Supplementary material

Table S1. Nutritional Composition of Equal Recipe Foods Extruded at High (HS), Medium (MS) and Low (LS) Shear.

Nutrient	HS	MS	LS
Moisture, %	8.69	9.87	10.40
Dry Matter, %	91.31	90.13	89.6
Organic Matter, %	86.03	85.15	84.1
Energy, kcal/kg	4652	4630	4541
Ash, %	5.28	4.98	5.50
Crude Protein, %	19.5	19.06	19.94
Fat Crude, %	15.04	14.94	14.28
Total Fatty Acids, %	13.95	13.75	13.08
Monounsaturated Fatty Acids, %	5.86	5.77	5.53
Polyunsaturated Fatty Acids, %	3.17	3.17	3.00
Saturated Fatty Acids, %	4.91	4.81	4.55
Total Fat as Triglycerides, %	14.60	14.38	13.68
Fiber Crude, %	1.30	1.2	1.10
Fiber Total Dietary, %	6.2	5.8	6.7
Fiber Insoluble, %	6.1	5.8	6.1
Fiber Soluble, %	< 0.2	< 0.2	0.6
Sucrose	1.03	1.07	0.83
Sugars - Total	1.67	1.07	0.83
Fructose	0.19	< 0.15	<0.15
Glucose	0.28	< 0.15	< 0.15
Lactose	0.17	< 0.15	<0.15
Maltose	<0.15	< 0.15	< 0.15
Starch	41.7	41.6	39.2
Resistant starch, %	0.650	0.940	1.057
Amino acids			
Alanine, %	1.40	1.36	1.41
Arginine, %	1.13	1.07	1.14
Aspartic Acid, %	1.50	1.46	1.56
Glutamic Acid, %	2.85	2.76	2.92
Glycine, %	1.66	1.59	1.71
Histidine, %	0.46	0.44	0.47
Isoleucine, %	0.70	0.68	0.70
Leucine, %	1.63	1.6	1.59
Lysine, %	1.17	1.13	1.24
Phenylalanine, %	0.82	0.8	0.79
Proline, %	1.49	1.4	1.39
Serine, %	0.77	0.75	0.80
Threonine, %	0.70	0.68	0.74
Tyrosine, %	0.42	0.4	0.37

Valine, %	0.89	0.84	0.88
Cystine, %	0.19	0.2	0.19
Methionine, %	0.37	0.38	0.39
Tryptophan, %	0.17	0.17	0.18
Taurine, %	0.10	0.11	0.10
Hydroxyproline, %	0.52	0.46	0.50
Minerals			
Chloride, %	0.42	0.42	0.45
Calcium, %	1.160	1.11	1.230
Magnesium, %	0.098	0.095	0.098
Phosphorus, %	0.86	0.84	0.90
Potassium, %	0.59	0.57	0.61
Sodium, %	0.173	0.175	0.188
Protein Crude, %	19.50	19.06	19.94
Sulfur, %	.22	0.22	.23
Chromium, ppm	0.26	0.33	0.24
Copper, ppm	17	17	19
Iron, %	0.0178	0.0177	0.0189
Manganese, ppm	19	20	20
Zinc, ppm	309.000	309	340.000
Molybdenum, ppm	0.25	0.3	0.23
Selenium, ppm	0.68	0.66	0.70
Aluminum, ppm	< 10.0	< 10.0	< 10.0
Cobalt, ppm	< 0.75	< 0.75	< 0.75
Nitrate, mg / kg	< 10	< 10	< 10
Vitamins			
Vitamin A, IU / 100 g	381.0	485	416.0
Vitamin B7 - Biotin, mg / 100 g	0.03	0.03	0.03
Choline, mg / 100 g	182	179	181
Vitamin B3 - Niacin, mg / 100 g	6.510	6.77	5.930
Vitamin B5 - Pantothenic Acid, mg / 100 g	1.240	1.15	1.200
Vitamin B9 - Folic Acid, mg / 100 g	0.141	0.117	0.108
Vitamin A - Beta Carotene, IU / 100 g	46.500	46.4	86.900
Vitamin A - Retinol, IU / 100 g	334.000	439	329.000
Vitamin B1 Mononitrate, mg / 100 g	2.81	2.61	2.73
Vitamin B1 Thiamin Base, mg / 100 g	2.58	2.4	2.51
VitaminB1-ThiamineHydrochloride, mg / 100 g	2.89	2.69	2.81
Vitamin B12 - Cobalamin, μg / 100 g	6.51	5.7	3.73
Vitamin B2 - Riboflavin, mg / 100 g	.62	0.619	.578
Vitamin B6 - Pyridoxine, mg / 100 g	0.8	0.8	0.9

Table S2. Analytical Methods of each Nutrient Analyzed in Equal Recipe Foods produced at High (HS), Medium (MS) and Low (LS) Shear, as well as Fecal Samples of Dogs Fed These Diets.

Analysis	Method
Acid Detergent Fiber QD002-Eurofins	ANKOM ADF for A2000 mod - Gravimetry
Aluminum by ICP	AOAC 984.27 mod,927.02 mod,985.01 mod,965.17 mod
Amino Acids Excluding Tryptophan	AOAC 982.30 mod.
Ash	AOAC 942.05
Biotin	Met. of Vitamin Assay,Interscience Publ.,Ch.12 - Nephelometry
Calories	Calorimetry
Chloride Soluble by Chloridometer	AOAC 2016.03, AOAC 971.27
Choline Total by Ion Chromatography	AOAC 2012.20 mod.
Chromium by ICP-MS - QD0K2	AOAC 2011.19 mod.
Cobalt by AAS	AOAC 965.17 / 968.08 modified
Cystine & Methionine	AOAC 994.12 mod.
Fat Crude by Acid Hydrolysis	AOAC 954.02
Fatty Acids-Omega 6 & 3 %W/W	AOCS Ce 2-66 mod., AOCS Ce 1b-89 mod.
Fiber Crude	AOAC 962.09; AOCS Ba 6-84
Fiber Dietary Complete	AOAC 991.43
Fiber Neutral Detergent	ANKOM NDF for A2000 mod. - Gravimetry
Fluoride	AOAC 975.08
Hydroxyproline - AOAC	AOAC 982.30 mod.
Macro Elements by ICP	AOAC 984.27 mod,927.02 mod,985.01 mod,965.17 mod
Minor Elements by ICP	AOAC 984.27 mod,927.02 mod,985.01 mod,965.17 mod
Moisture - Forced Draft Oven	AOAC 930.15
Molybdenum by ICP-MS - QD0K1	AOAC 2011.19 mod.
Niacin or Niacinamide - AOAC	AOAC 944.13 mod.
Nitrate by IC	Internal Method based on EN 12014-2, (QA02F) J. AOAC Int., 2005, 88(6), 1793-1796 - IC-EC
Nitrite as Nitrogen - AOAC 968.07	AOAC 968.07
Pantothenic Acid - AOAC	AOAC 945.74 (mod.)
Protein by Kjeltec (Kjeldahl Replacement)	AOAC 2001.11
Selenium by ICP-MS - QD0K0	AOAC 2011.19 mod.
Starch Total - AOAC	AOAC 996.11
Sugar Profile by HPLC - AOAC	AOAC 982.14, mod.
Sulfate - AOAC Gravimetric	AOAC 920.46
Sulfur by ICP	Internal Method - MET3289 - ICP-OES
Taurine Food	AOAC 982.30 mod.
Total Folate as Folic Acid	AOAC 992.05 mod.
Tryptophan - AOAC	AOAC 988.15 mod.
Vitamin A Total-AOAC	AOAC 974.29 Mod.
Vitamin B1	AOAC 942.23 mod.
Vitamin B12 - AOAC	AOAC 952.20 mod.
Vitamin B2	AOAC 970.65 mod.
Vitamin B6 -Pyridoxine by HPLC	J. AOAC 88, 30-37 (2005), mod.
Water Activity	AOAC 978.18 mod.

Table S3. Estimated mean [95% confidence interval] for blood chemistry of dogs fed diets produced at high, medium and low shear (HS, MS and LS, respectively).

**Parameter**	**HS**	**MS**	**LS**	***P***	**Reference range**
ALT, U/L	24.8 [21.4, 29.4]	25.6 [22.0, 30.6]	26.8 [22.9, 32.4]	0.0822	17 – 55
Albumin, g/dL	3.34 [3.10, 3.59]	3.35 [3.12, 3.59]	3.36 [3.13, 3.59]	0.9098	2.8- 4.0
Alb:Glob	1.66 [1.44, 1.88]	1.62 [1.40, 1.84]	1.64 [1.42, 1.86]	0.7102	1.1- 2.4
ALP, U/L	60.3 [51.7, 68.9]	61.0 [52.4, 69.6]	55.3 [46.7, 63.9]	0.0580	17- 134
BUN, mg/dL	12.6[b] [11.0, 14.9]	12.8[ab] [11.0, 15.3]	14.2[a] [12.1, 17.2]	0.0284	7.6-19.3
BUN:creat	14.8[b] [13.2, 16.6]	15.0[b] [13.4, 16.8]	16.4[a] [14.7, 18.4]	0.0040	11.3- 26.4
Creatinine, mg/dL	0.864 [0.771, 0.963]	0.862 [0.768, 0.962]	0.875 [0.786, 0.970]	0.4778	0.5- 1.0
Calcium, mg/dL	9.95 [9.81, 10.08]	9.99 [9.86, 10.13]	10.02 [9.89, 10.16]	0.3718	9.0- 10.8
Chloride, mmol/L	115.8 [114.8, 116.8]	116.4 [115.5, 117.2]	116.4 [115.4, 117.5]	0.2253	108- 116
Cholesterol, mg/dL	191.3 [173.9-210.5]	191.7 [174.2-210.9]	188.7 [171.5-207.6]	0.7233	127- 318
Globulin, g/dL	2.03 [1.83, 2.23]	2.06 [1.86, 2.26]	2.07 [1.87, 2.27]	0.7233	1.5- 2.6
Glucose, mg/dL	94.0 [90.6, 97.3]	93.7 [90.3, 97.0]	93.6 [90.2, 96.9]	0.9717	79- 116
Phosphorous, mg/dL	3.42 [3.27, 3.57]	3.39 [3.25, 3.54]	3.45 [3.31, 3.60]	0.8077	2.3- 4.7
Magnesium, mg/dL	1.83 [1.74, 1.92]	1.82 [1.73, 1.92]	1.85 [1.76, 1.93]	0.7026	1.7- 2.2
Potassium, mmol/L	4.68 [4.59, 4.77]	4.67 [4.58, 4.76]	4.75 [4.66, 4.83]	0.2153	3.7- 5.1
Sodium, mmol/L	147.5 [145.7, 149.3]	147.8 [145.9, 149.6]	148.0 [146.2, 149.8]	0.2317	145- 150
Na:K	31.5 [30.9, 32.2]	31.7 [31.0, 32.3]	31.2 [30.6, 31.9]	0.3068	29- 40
Total protein, g/dL	5.43 [5.29, 5.56]	5.46 [5.29, 5.64]	5.48 [5.33, 5.63]	0.5898	4.8- 6.1
Triglycerides, mg/dL	49.7 [39.5, 62.4]	50.5 [40.2, 63.5]	49.0 [39.0, 61.6]	0.8934	19- 119

[ab] Different letters indicate treatment differences (P < 0.05)

Table S4. Estimated mean [95% confidence interval] for complete blood count (CBC) of dogs fed diets produced at high, medium and low shear (HS, MS and LS, respectively).

Parameter	HS	MS	LS	*P*	Reference range
IRF, %	15.6 [12.9, 18.4]	16.3 [13.5, 19.0]	18.0 [15.2, 20.7]	0.2455	5.2- 32.7
MCH, pg	23.1 [22.8, 23.5]	23.1 [22.7, 23.5]	23.2 [22.8, 23.6]	0.8617	21.6- 24.6
MCHC, g/dL	34.8 [34.6, 35.1]	34.8 [34.6, 35.1]	34.9 [34.7, 35.2]	0.4726	32.4- 36.0
MCV, fL	66.7 [65.6, 67.37]	66.7 [65.6, 67.7]	66.6 [65.5, 67.7]	0.8014	63.6- 72.9
Platelets, k/μL	316.0 [269.5, 370.5]	309.2 [263.7, 362.5]	306.3 [261.2, 359.0]	0.4954	131- 429
HCT, %	48.1 [46.8, 49.5]	47.9 [46.5, 49.3]	48.8 [47.4, 50.2]	0.2894	35.2- 54.2
Red Blood Cells, M/μL	7.24 [6.96, 7.53]	7.20 [6.92, 7.49]	7.35 [7.06, 7.63]	0.2690	5.16- 8.24
RDW, fL	34.0 [33.2, 34.7]	34.0 [33.3, 34.7]	33.9 [33.2, 34.6]	0.8014	31.4- 37.0
HGB, g/dL	16.8 [16.3, 17.2]	16.7 [16.2, 17.1]	17.0 [16.6, 17.5]	0.2197	11.9- 18.5
Reticulocytes, %	0.695 [0.561, 0.828]	0.677 [0.573, 0.781]	0.704 [0.555, 0.852]	0.9226	0.23- 1.30
Basophils, %	0.206 [0.163, 0.250]	0.217 [0.172, 0.262]	0.214 [0.147, 0.281]	0.9185	-
Eosinophils, %	3.41 [2.62, 4.45]	3.14 [2.41, 4.10]	3.12 [2.39, 4.07]	0.4514	-
Lymphocytes, %	30.8 [27.7, 33.9]	30.2 [27.1, 33.3]	30.0 [26.9, 33.1]	0.8562	-
Monocytes, %	5.07 [4.52, 5.62]	4.92 [4.37, 5.47]	5.56 [5.01, 6.11]	0.0642	-
Neutrophils, %	60.2 [55.4, 65.0]	61.1 [56.3, 65.9]	60.7 [55.9, 65.5]	0.8202	-
Reticulocytes, M/μL	0.065 [0.054, 0.076]	0.060 [0.049, 0.071]	0.062 [0.052, 0.073]	0.4785	-
Eosinophils, k/μL	0.215 [0.141, 0.326]	0.198 [0.138, 0.285]	0.198 [0.135, 0.292]	0.5285	0.06- 0.62
Lymphocytes, k/μL	1.90 [1.54, 2.26]	1.87 [1.47, 2.26]	1.88 [1.49, 2.28]	0.8059	0.87- 3.04
Monocytes, k/μL	0.354 [0.303, 0.405]	0.346 [0.295, 0.396]	0.319 [0.269, 0.370]	0.2912	0.14- 0.69
Neutrophils, k/μL	3.84 [3.45, 4.27]	3.86 [3.47, 4.29]	3.77 [3.40, 4.19]	0.9317	2.09- 7.32
Basophils, k/μL	0.012 [0.005, 0.0189]	0.016 [0.009, 0.0226]	0.013 [0.0053, 0.0198]	0.7036	0.00- 0.04
White Blood Cells, k/μL	6.65 [5.90, 7.40]	6.53 [5.80, 7.26]	6.40 [5.66, 7.13]	0.7463	3.3- 11.3

Table S5. Estimated mean [95% confidence interval] for fecal minerals of dogs fed diets produced at high, medium and low shear (HS, MS and LS, respectively).

**Fecal mineral**	**HS**	**MS**	**LS**	***P***
Calcium, %	7.58[ab] [7.01, 8.14]	7.17[b] [6.60, 7.73]	8.01[a] [7.44, 8.57]	0.0622
Phosphorous, %	4.21 [3.85, 4.57]	4.21 [3.90, 4.53]	4.49 [4.02, 4.95]	0.4365
Potassium, %	0.362 [0.300, 0.424]	0.370 [0.306, 0.434]	0.359 [0.296, 0.422]	0.9235
Magnesium, %	0.537 [0.483, 0.590]	0.530 [0.476, 0.583]	0.542 [0.488, 0.596]	0.9204
Sodium, %	0.257[a] [0.213, 0.310]	0.208[ab] [0.172, 0.252]	0.182[b]; 0.154-0.215	0.0123
Zinc, ppm	2,040 [1,897, 2,183]	2,008 [1,865, 2,152]	2,160 [2,016, 2,303]	0.1934
Copper, ppm	99.9 [92.6, 108.5]	95.7 [88.9, 103.5]	101.8 [94.2, 110.7]	0.3925
Iron, ppm	1,226 [1,110, 1,369]	1,190 [1,122, 1,266]	1,259 [1,181, 1,349]	0.4399
Manganese, ppm	146.5 [129.0, 164.0]	138.8 [125.2, 152.5]	160.6 [142.2, 179.0]	0.1178

[ab]Different letters indicate treatment differences ($P < 0.05$).

Table S6. Estimated means (+- standard errors) for fecal amino acids concentrations of dogs fed diets produced at high, medium and low shear (HS, MS and LS, respectively).

**Analyte**	**HS**	**MS**	**LS**	***P***	**q-value**
alanine	-0.134 ± 0.2106	0.016 ± 0.2106	-0.091 ± 0.2216	0.612	0.518
arginine	-0.087 ± 0.5565	0.37 ± 0.5565	-0.094 ± 0.5798	0.352	0.411
asparagine	0.358 ± 0.7919	0.982 ± 0.7919	0.588 ± 0.8262	0.494	0.477
aspartate	-0.209 ± 0.4044	-0.04 ± 0.4044	-0.519 ± 0.4213	0.197	0.316
cysteine	-0.02 ± 0.1613	0.116 ± 0.1625	-0.005 ± 0.1697	0.219	0.33
glutamate	-0.420[b] ± 0.3304	0.055[a] ± 0.3307	-0.269[ab] ± 0.3436	0.041	0.135
glutamine	0.073 ± 0.3285	0.213 ± 0.3289	0.217 ± 0.3434	0.717	0.56
glycine	0.086b ± 0.2516	0.470a ± 0.2519	0.303ab ± 0.263	0.051	0.142
histidine	-0.014 ± 0.3612	0.453 ± 0.3612	0.047 ± 0.3753	0.097	0.217
isoleucine	-0.078 ± 0.2812	0.144 ± 0.2812	-0.115 ± 0.2939	0.348	0.41
leucine	-0.164 ± 0.2832	0.05 ± 0.2832	-0.177 ± 0.2962	0.428	0.44
lysine	-0.224 ± 0.2531	-0.064 ± 0.2531	-0.224 ± 0.2637	0.543	0.487
methionine	0.061 ± 0.2062	0.164 ± 0.2062	0.023 ± 0.2158	0.597	0.514
phenylalanine	-0.153 ± 0.2875	-0.068 ± 0.2875	-0.185 ± 0.2978	0.783	0.581
proline	-0.098 ± 0.2248	0.184 ± 0.2251	-0.012 ± 0.2369	0.158	0.286
serine	-0.081 ± 0.3433	0.104 ± 0.3433	-0.275 ± 0.3563	0.22	0.33
taurine	0.004 ± 0.8388	-0.264 ± 0.8388	-0.056 ± 0.8522	0.707	0.558
threonine	-0.173[b] ± 0.2421	0.234[a] ± 0.2424	0.006[ab] ± 0.2551	0.047	0.142
tryptophan	-0.347 ± 0.3363	-0.202 ± 0.3363	-0.196 ± 0.3527	0.774	0.581
tyrosine	-0.132 ± 0.3091	-0.134 ± 0.3091	-0.34 ± 0.3236	0.539	0.485
valine	-0.21 ± 0.3181	0.036 ± 0.3181	-0.258 ± 0.3335	0.38	0.417

[ab]Different letters indicate treatment differences ($P < 0.05$).

Table S7. Estimated means (+- standard errors) for fecal di- and tripeptides metabolomics of dogs fed diets produced at high, medium and low shear (HS, MS and LS, respectively).

Analyte	HS	MS	LS	*P*	q-value
ala-ile-ala	-0.221 ± 0.3322	0.175 ± 0.3322	0.089 ± 0.3505	0.262	0.367
ala-leu-ala	-0.275 ± 0.3328	0.142 ± 0.3328	0.084 ± 0.3532	0.245	0.352
alanylleucine	-0.006 ± 0.2777	0.044 ± 0.2777	0.177 ± 0.2932	0.67	0.545
glycylisoleucine	0.123 ± 0.3494	0.157 ± 0.3469	-0.052 ± 0.365	0.682	0.551
glycylleucine	-0.109 ± 0.2561	-0.103 ± 0.2546	-0.082 ± 0.265	0.986	0.644
glycylvaline	-0.108 ± 0.2611	-0.032 ± 0.2611	-0.104 ± 0.2744	0.901	0.623
histidylalanine	0.136 ± 0.3402	0.187 ± 0.3402	0.019 ± 0.3578	0.786	0.582
isoleucylglycine	0.259 ± 0.2421	0.013 ± 0.2421	-0.053 ± 0.2516	0.111	0.230
leucylalanine	-0.048 ± 0.3119	0.046 ± 0.3119	0.24 ± 0.3254	0.376	0.417
leucylglutamine*	0.059 ± 0.2949	0.302 ± 0.2949	0.376 ± 0.3088	0.281	0.374
leucylglycine	0.025 ± 0.2296	-0.098 ± 0.2296	0.073 ± 0.239	0.500	0.477
lysylleucine	0.068 ± 0.2676	0.154 ± 0.2676	0.188 ± 0.2792	0.786	0.582
phenylalanylalanine	-0.045[b] ± 0.3102	0.359[ab] ± 0.3102	0.565[a] ± 0.3254	0.026	0.110
phenylalanylglycine	-0.078 ± 0.2536	0.054 ± 0.2536	0.134 ± 0.2662	0.501	0.477
prolylglycine	-0.322 ± 0.3687	0.291 ± 0.3687	0.115 ± 0.3903	0.101	0.218
threonylphenylalanine	-0.241 ± 0.4171	0.07 ± 0.4171	0.115 ± 0.4317	0.306	0.393
tryptophylglycine	0.012[b] ± 0.207	0.158[ab] ± 0.207	0.364[a] ± 0.2155	0.041	0.135
tyrosylglycine	-0.049 ± 0.2617	0.138 ± 0.2617	0.12 ± 0.2751	0.559	0.495
val-val-ala	-0.064 ± 0.2724	0.011 ± 0.2724	0.029 ± 0.285	0.870	0.611
valylglutamine	-0.036 ± 0.3136	0.117 ± 0.312	0.074 ± 0.3237	0.716	0.560
valylglycine	0.136 ± 0.1857	-0.019 ± 0.1846	0.02 ± 0.1926	0.432	0.441
valylleucine	-0.01 ± 0.2952	-0.045 ± 0.2952	0.004 ± 0.3093	0.970	0.640

[ab]Different letters indicate treatment differences ($P < 0.05$).

Table S8. Estimated means (+- standard errors) for putrefactive compounds metabolomics of dogs fed diets produced at high, medium and low shear (HS, MS and LS, respectively).

Analyte	Estimate HS	Estimate MS	Estimate LS	*P*	q-value
3-indoleglyoxylic acid	$-0.223^{b} \pm 0.2357$	$-0.201^{b} \pm 0.2357$	$0.148^{a} \pm 0.2403$	0.001	0.042
3-hydroxyindolin-2-one	$-0.560^{a} \pm 0.3994$	$-1.357^{b} \pm 0.3994$	$-1.279^{b} \pm 0.4122$	0.002	0.042
indolepropionate	$-0.283^{b} \pm 0.6058$	$0.436^{a} \pm 0.6055$	$0.223^{ab} \pm 0.6145$	0.008	0.068
indole	$0.233^{a} \pm 0.4204$	$-0.601^{b} \pm 0.4204$	$-0.434^{b} \pm 0.4379$	0.01	0.073
phenol sulfate	$0.413^{a} \pm 0.6018$	$-0.440^{b} \pm 0.6018$	$-0.264^{ab} \pm 0.6158$	0.014	0.087
indolin-2-one	$0.450^{a} \pm 0.8927$	$-0.917^{b} \pm 0.8927$	$-0.375^{ab} \pm 0.9153$	0.015	0.09
2-hydroxy-3-methylvalerate	$-0.980^{b} \pm 0.6032$	$0.201^{a} \pm 0.6032$	$0.025^{ab} \pm 0.6337$	0.02	0.095
4-methyl-2-oxopentanoate	$-0.397^{ab} \pm 0.4071$	$-0.382^{a} \pm 0.4071$	$-0.975^{b} \pm 0.4208$	0.025	0.109
indoleacetylglycine	$-1.012^{b} \pm 0.4675$	$-0.323^{a} \pm 0.4675$	$-0.588^{ab} \pm 0.4826$	0.044	0.138
alpha-hydroxyisocaproate	-1.173 ± 0.7001	-0.124 ± 0.7001	-0.198 ± 0.7325	0.063	0.164
indolelactate	-0.278 ± 0.6927	1.068 ± 0.6927	0.636 ± 0.7386	0.068	0.17
imidazole lactate	-0.039 ± 0.5375	0.857 ± 0.5375	0.454 ± 0.5643	0.077	0.185
alpha-hydroxyisovalerate	-1.244 ± 0.606	-0.414 ± 0.606	-0.415 ± 0.6343	0.085	0.197
5-hydroxyindoleacetate	-0.195 ± 0.3977	-0.47 ± 0.3977	-0.528 ± 0.4045	0.111	0.23
phenyllactate (PLA)	0.051 ± 0.543	0.825 ± 0.543	0.602 ± 0.5684	0.118	0.24
4-hydroxyphenylpyruvate	0.022 ± 0.4193	0.072 ± 0.4193	-0.59 ± 0.4507	0.149	0.273
3-methyl-2-oxovalerate	-0.523 ± 0.4792	-0.473 ± 0.4792	-0.937 ± 0.4949	0.196	0.316
indoleacetate	-0.027 ± 0.1846	-0.047 ± 0.1845	-0.219 ± 0.1923	0.272	0.369
indoleacrylate	-0.175 ± 0.3578	-0.347 ± 0.3578	-0.502 ± 0.3694	0.292	0.38
3-methyl-2-oxobutyrate	-0.486 ± 0.3857	-0.494 ± 0.3857	-0.764 ± 0.3975	0.346	0.409
phenylpyruvate	-0.131 ± 0.3602	-0.117 ± 0.3602	-0.431 ± 0.3771	0.372	0.417
3-formylindole	-0.266 ± 0.1975	-0.235 ± 0.1978	-0.391 ± 0.2088	0.443	0.449
3-(4-hydroxyphenyl) propionate	-0.078 ± 0.4893	0.127 ± 0.4896	0.007 ± 0.4989	0.614	0.519
indole-3-carboxylate	-0.251 ± 0.3334	-0.413 ± 0.3334	-0.396 ± 0.3499	0.758	0.578
2-oxindole-3-acetate	0.065 ± 0.2847	-0.028 ± 0.2847	0.082 ± 0.2937	0.768	0.58

4-hydroxyphenylacetate	0.116 ± 0.2998	0.229 ± 0.2998	0.133 ± 0.3101	0.791	0.583
phenylacetate	0.255 ± 0.5457	0.278 ± 0.5457	0.347 ± 0.5647	0.958	0.637

[ab]Different letters indicate treatment differences ($P < 0.05$).

Table S9. Fecal advanced glycation end-products (AGEs) Metabolomics of Dogs (N=24) fed Diets produced at High, Medium and Low Shear (HS, MS and LS, respectively).

**Analyte**	**Estimate HS**	**Estimate MS**	**Estimate LS**	***P***	**q-value**
carboxy-methyl-arginine	-0.810 ± 0.423	-0.811 ± 0.423	-0.559 ± 0.4309	0.298	0.384
N6-carboxyethyllysine	-0.245 ± 0.265	-0.219 ± 0.265	-0.06 ± 0.2713	0.31	0.394
N6-carboxymethyllysine	-0.267 ± 0.2084	-0.524 ± 0.2087	-0.488 ± 0.2193	0.137	0.261
pyrraline	-0.109[b] ± 0.2966	0.380[a] ± 0.2966	0.478[a] ± 0.305	0.001	0.042

[ab]Different letters indicate treatment differences ($P < 0.05$).
